# Post-mastectomy Pain Syndrome: A Review Article and Emerging Treatment Modalities

**DOI:** 10.7759/cureus.56653

**Published:** 2024-03-21

**Authors:** Jay D Shah, Kennedy Kirkpatrick, Krishna Shah

**Affiliations:** 1 Anesthesiology, Baylor College of Medicine, Houston, USA; 2 Anesthesiology and Interventional Pain, Baylor College of Medicine, Houston, USA

**Keywords:** pain control after mastectomy, chronic breast pain, neuromodulation, breast cancer pain, pmps, post mastectomy pain syndrome

## Abstract

Post-mastectomy pain syndrome (PMPS) is a syndrome broadly applied to the development of chronic pain after surgical breast intervention (i.e., lumpectomy and mastectomy). The incidence of PMPS is likely underreported, and this has contributed to a paucity of high-level evidence related to the treatment of the aforementioned condition. A drive to reduce the burden of opioid use has led to pain management physicians trialing a variety of strategies to help patients manage PMPS. This review discusses the latest evidence behind treatment options for PMPS, exploring medications as well as interventional techniques (e.g., nerve blocks, radiofrequency ablation, neuromodulation, and intrathecal drug delivery systems). Recent advances in neuromodulation technology are of particular interest here due to the well-localized nature of PMPS-related pain and the specificity with which modern neuromodulation techniques can generate an effect. Finally, the review proposes a framework with which to approach the care of patients with PMPS, with a specific emphasis on the early consideration of neuromodulation techniques along with functional and physical therapy to reduce patient medication burden and improve overall quality of life.

## Introduction and background

Breast cancer is the most common cancer diagnosed globally according to the American Cancer Society, and one in eight women will develop invasive breast cancer throughout their life [1]. It was estimated that 287,850 new cases of invasive breast cancer were expected to be diagnosed along with 43,250 deaths from breast cancer in the United States in 2022 alone [1]. Treatment for breast cancer is dependent on staging, with treatment modalities ranging from surgical excision with mastectomy or lumpectomy with radiation, chemotherapy, immunotherapies, endocrine therapies, or a combination of the aforementioned [2]. Surgical excision is a cornerstone of breast cancer treatment; however, it does not come without complications. Postoperative pain extending beyond three months following mastectomy is experienced among 36%-47% of patients [2-4].

Chronic pain following mastectomy, referred to at that time as intercostobrachial nerve entrapment syndrome, was first identified in a case series of patients who underwent mastectomy in the 1970s [2]. This condition is now referred to as post-mastectomy pain syndrome (PMPS). The International Association for the Study of Pain (IASP) defines PMPS as persistent, neuropathic pain that develops soon after mastectomy or lumpectomy located in the anterior surface of the chest axilla, shoulder, or upper half of the arm [2]. Although the timeline to diagnose PMPS is widely debated with three to six months of pain being generally agreed upon, a recent systematic review suggested that six months of neuropathic pain that occurs at least 50% of the time should be used for PMPS research to create uniformity in future studies [5,6]. With improvement and compliance in breast cancer screening, leading to earlier diagnosis, and innovation in treatment, breast cancer prognosis has significantly improved with current five-year survival rates near 90% and 10-year survival near 80% [2]. As more women are diagnosed earlier and survival rates following mastectomies increase, the risk of developing PMPS increases, which currently has no agreed-upon first-line treatment.

The most recent retrospective analysis to quantify PMPS (defined by pain for at least three months) was the largest cohort to date. It estimated the prevalence of PMPS in breast cancer patients who underwent surgical management at 28.2% [3]. PMPS not only affects functional mobility but also has a severe impact on the quality of life. As the pathophysiology of PMPS is poorly understood, the options for treatment modalities range broadly with no current gold standard. New treatment modalities have emerged in the past decade that have increased the options available to PMPS patients with better control of chronic pain. A systematic review in 2020 briefly highlights some of the new treatment modalities including peripheral nerve blocks, a concise review of certain oral medications, and neuromodulation [3,4]. However, research involving neuromodulation, oral medications, topical medications such as capsaicin, and infusions with drugs like ketamine has evolved since the last comprehensive review on PMPS treatment modalities. This review article seeks to describe advances in PMPS management, highlight new data in the literature surrounding existing treatment modalities, and analyze the outcomes of current literature on PMPS treatment to create an algorithm for providers to use.

Pathophysiology

Although the pathophysiology of PMPS is not fully understood, the most common theory agreed upon in literature is neuralgia of the intercostobrachial nerve [3,4] The intercostobrachial nerve is the lateral cutaneous branch of the second intercostal nerve, which travels through the serratus anterior muscle and reaches the axilla and inner arm area to provide cutaneous sensation. Dissection of axillary nodes or manipulation from retraction during mastectomy can lead to inflammation and nerve injury. Nerve injury can then sensitize peripheral nociceptors leading to ectopic neural activity, resulting in sensitivity to external stimuli and sensation of pain [3,4].

## Review

Pharmacologic management

Oral Medications

The use of pharmacologic therapy has long been considered a first-line approach along with physical therapy for a multitude of chronic pain syndromes, including cancer-related pain and specifically for post-mastectomy pain [5]. The traditional sequence of therapy includes the use of non-steroidal anti-inflammatory medications (NSAIDs) followed by opioids for a variety of cancer-related pain syndromes. Additional medications that have shown significant promise for PMPS include antidepressants (selective serotonin reuptake inhibitors [SSRI], serotonin and norepinephrine reuptake inhibitors [SNRI], and tricyclic antidepressants [TCAs]), gabapentinoids (gabapentin and pregabalin), and a few other non-opioid medications that are now gaining traction [3,6].

Antidepressants

SSRI/SNRI as well as TCAs are a mainstay of PMPS treatment, specifically when the pain symptoms are neuropathic in nature. TCAs primarily work by inhibiting monoamine receptors at the presynaptic terminal. Inhibition of noradrenaline via a2 receptors in the dorsal horn of the spinal cord has also been implicated as a major driver of pain relief in these patient populations. Multiple studies have investigated the utility of these classes of medication in the treatment of PMPS [7-9]. One such double-blind, crossover trial with a two-week washout on 15 patients evaluating the use of TCAs in PMPS showed >50% pain reduction at the two- and four-week mark after initiation of TCA (amitriptyline) [8]. Another study evaluating 15 patients on venlafaxine (SNRI) also showed a statistically significant improvement in PMPS-related neuropathic pain, with 11/15 patients showing >50% improvement in pain relief [9]. There was a dose-dependent relationship on pain relief as well, with high-dose venlafaxine showing a significantly lower pain burden than in lower doses. Another trial showed that initiation of venlafaxine in the perioperative period (i.e., the night before surgery) significantly reduced the incidence of PMPS in the six-month postoperative period [7]. The major concern regarding TCAs and SNRIs is the ability of patients to tolerate them, especially when looking at TCAs. In the aforementioned study regarding amitriptyline, >50% of patients eventually stopped taking the medication due to adverse events. Common side effects include anticholinergic symptoms, sex drive changes, and fluctuations in weight, all of which may prove a barrier to patients wishing to manage their PMPS symptoms.

Gabapentinoids

Another mainstay of treatment for patients with PMPS (specifically the neuropathic manifestations) is the gabapentinoid medications: pregabalin and gabapentin. These medications work by modulating glutamate release (via calcium) from activated pain neurons, thus attenuating central sensitization and inhibiting pain transmission. Multiple studies have shown the utility of gabapentin in PMPS, showing >50% pain relief at the four-week mark, with successful relief, and improvement in the quality of life through a three-month follow-up period as well [7,10]. One study evaluated a multimodal analgesia regimen of gabapentin, NSAIDs, and morphine together, and it showed significant improvement in initial pain relief when compared to gabapentin alone or gabapentin with just NSAIDs [6,10]. In all three groups, there was >50% pain relief sustained from the initial two-week follow-up till the six-week endpoint of the study. Interestingly, however, there was no difference in long-term pain relief among all three groups, although they were all successful in significantly alleviating PMPS-related pain. Another study evaluating pregabalin in 35 patients showed significantly reduced visual analog scale (VAS) pain scores as well as significantly improved quality of life at the one- and two-month follow-up marks [11,12].

Memantine

Another medication that may have a role in post-mastectomy pain prophylaxis is memantine, an N-methyl-D-aspartate (NMDA) antagonist that is primarily known for its use in the management of Alzheimer’s disease [13]. It has been hypothesized to have advantageous effects in modulating pain signaling within the excitatory pathways of afferent neurons as NMDA antagonism leads to a decrease in sustained neuronal depolarization. A recent promising randomized controlled trial conducted in mastectomy patients had groups taking a four-week course of memantine starting two weeks before the operation [14]. It showed that at the three-month postoperative mark, the memantine group had significantly less pain than the control group. Furthermore, patients who took memantine before surgery were 1/6th as likely to require any medical management for neuropathic pain in the long-term postoperative period. However, there was no significant difference in pain intensity or overall quality of life at the six-month postoperative period. As this was just a pilot trial, further studies are recommended and underway to elucidate the perioperative utility of memantine in the prevention and possible treatment of chronic post-mastectomy pain.

Topical medications

Topical Capsaicin

Capsaicin, a naturally occurring alkaloid found in chillies, is a TRPV1 antagonist that depletes substance P in small fiber neurons, thereby driving the attenuation of pain signals and transmission. Topical capsaicin has long been used to treat pain and its utility in post-mastectomy pain has been investigated since the 1980s with highly encouraging results [15]. The earliest trials with 0.025% capsaicin found improvement in PMPS symptoms by the four-week mark with >50% pain relief found in a majority of patients at the six-month follow-up period as well. Another study of 0.075% capsaicin found >50% pain relief at the six-month mark via the VAS scale for 62% of patients in the trial [15]. Topical capsaicin works well as an adjunct therapy to oral medications as well as recent case reports have shown the utility of topical capsaicin in achieving near-total pain relief in patients who were not fully responding to gabapentinoids and TCAs. Another recent case report involved the use of an 8% capsaicin patch and found that in addition to oral pain medications (pregabalin), near-total pain relief could be achieved. While topical capsaicin may have utility as a standalone treatment option, it shows great promise as part of combination therapy as well.

Interventional techniques

Electrocutaneous Therapy

The utilization of electrical stimulation to disrupt the firing patterns of nerves has been well studied in the chronic pain landscape, and many invasive and noninvasive techniques (discussed here) have shown promise in the long-term management of chronic pain syndromes. One of the noninvasive techniques that has shown particular promise in PMPS is scrambler therapy, an FDA-cleared treatment for neuropathic pain. Scrambler therapy involves placing electrodes in areas where patients are experiencing pain and delivering 16 different synthesized waveforms resembling c-fiber action potentials to surface receptors [16,17]. This activates Na and Ca channels in the painful area with an end goal of resetting the firing patterns of the c-fibers causing "scrambling" of signals sent to the brain, leading to higher transmission of non-pain signals along the original chronic pain pathways. A randomized clinical trial evaluating the efficacy of scrambler therapy in chronic refractory neuropathic pain (e.g., post-herpetic neuralgia and post-surgical pain) found that the group of patients receiving scrambler therapy found 91% improvement in pain scores and functionality when compared to traditional medical therapy. Allodynia decreased, and the overall usage of opioids decreased by 75% compared to the control group [16].

While the use of scrambler therapy in PMPS has not been fully established, multiple trials have shown the utility of this therapy in chronic neuropathic pain syndromes. A recent case series around the use of scrambler therapy in PMPS in three patients found significant improvement in pain scores and functionality across the board, with the elimination of chronic opioid use in one of the three patients. One of the patients, who was experiencing significant allodynia post lumpectomy (redness, edema, swelling, loss of function, and burning pain) had near-total symptomatic and pain relief with no recurrence of symptoms during the follow-up period. There is currently a significant amount of active research for this promising therapy as highlighted by a recent *NEJM *review of various electrocutaneous modalities for chronic pain [17]. While this therapy has been around for almost 20 years, it is continuing to gain more and more traction as a noninvasive, interventional approach that can significantly reduce the burden of opioid medications in our chronic pain patients.

Trigger Point Injections

A simple yet effective procedure that has great promise in the treatment algorithm for post-mastectomy pain is trigger point injections. This is especially true in patients who are experiencing point tenderness and neuropathic pain symptoms originating from the inframammary folds (at the site of T4 and T5 intercostal nerve branches.) As one of the hypothesized causes of PMPS is peripheral nerve injury during surgery (specifically at the T4/T5 levels) and subsequent neuroma and hypersensitivity, the idea of a trigger point injection with perineural infiltration of local anesthesia and steroid is mechanistically sound. In a recent study, PMPS-related neuropathic pain was treated with trigger point injections consisting of 2 mL 1:1 0.5% bupivacaine and 4 mg/mL dexamethasone. A successful injection was defined as sustained pain relief at and beyond the three-month mark. About 92% of injections met the success criteria with 72% of patients only needing a single injection for sustained, long-term pain relief (mean follow-up was 44 months) [18]. These extremely encouraging results underlie the importance of trigger point injections in the PMPS algorithm, especially when a physical exam can readily identify a neuroma site and an injection can be quickly, safely, and effectively administered.

Regional Anesthesia

Regional anesthesia using peripheral nerve blockade is important in the management of PMPS, especially when considering the perioperative management of patients undergoing mastectomy. Nerves thought to trigger PMPS pain can be blocked using regional anesthesia and have shown tremendous benefit as an adjunctive treatment in decreasing PMPS pain in current literature. A variety of blocks have shown promise in PMPS, including intercostobrachial nerve blocks, thoracic paravertebral blocks, etc.; however, the one that has lately been gaining the most traction is the stellate ganglion block (SGB).

SGB is a peripheral nerve block of the cervical sympathetic chain that affects the ipsilateral head, neck, upper extremity, and upper thorax. Mechanistically, SGB is thought to inhibit sympathetic nervous system (SNS) activity, thus decreasing the chronic stress responses in various disease processes [19]. Newer theories suggest a relationship with nerve growth factor (NGF), which is involved in a variety of signaling events related to acute and chronic stress [19,20]. Data is limited in PMPS treatment, although few cases render it an effective treatment modality. One study compared classic anterior versus oblique SGB for the treatment of PMPS and found that while both approaches lowered pain scores, decreased opioid consumption, and decreased areas of allodynia, the oblique approach was deemed safer and achieved higher patient satisfaction [19]. Patients still required morphine for pain control suggesting incomplete blockade of the upper limb; however, the pain remained decreased three months after treatment at vertebral levels T1-T4, suggesting effective analgesia at higher vertebral levels [19]. The only other study of PMPS treatment with SGB emphasized the efficacy of SGB with bupivacaine plus ketamine compared to bupivacaine alone or bupivacaine with morphine [21]. In this study, the bupivacaine plus ketamine group demonstrated decreased pain scores up to three months following the procedure, improved mobility, and decreased need for other analgesic drugs [21].

SGB has recently been trialed for use in patients with anosmia, long-COVID, menopause, and even ventricular arrhythmias with promising results. It has also shown effectiveness in chronic pain syndromes such as complex regional pain syndrome (CRPS). There is a paucity of literature on the use of SGB for PMPS, but given the pathophysiology of PMPS, it should be considered in the treatment algorithm for both diagnostic and therapeutic relief [22,23].

Radiofrequency Ablation

Radiofrequency ablation (RFA) has been used for the treatment of chronic pain conditions for decades including cervical and lumbar back pain, trigeminal and occipital neuralgia, and various other pain syndromes resistant to standard treatment modalities like tricyclic antidepressants, amitriptyline, SNRIs, duloxetine, and gabapentinoids [24-30]. Generally, for RFA treatment, under fluoroscopic guidance, radiofrequency (RF) waves are delivered through an electrode that is placed near the target site to disrupt the transmission of pain impulses delivered by the nerve [24].

Originally, continuous radiofrequency (CRF) was the only radiofrequency modality available. CRF creates sympathetic denervation by delivering uninterrupted RF current at temperatures ranging from 55°C to 80°C depending on location [24,25]. Cycling between on and off currents, once a target temperature is reached, is done to maintain a predetermined set point to destroy nerve fibers at the target area, thus decreasing nerve impulses and pain sensation [24].

Pulsed radiofrequency (PRF) was developed as a less destructive option compared to CRF after a randomized controlled trial (RCT) using different temperatures showed no statistical difference in the outcomes, suggesting that maybe the electrical current as opposed to temperature leads to clinical benefits [30]. Due to its higher safety profile and clinical efficacy, PRF has grown in popularity and utility over conventional radiofrequency ablation. PRF uses lower temperatures and higher voltage currents in a pulsatile manner to deliver enough RF energy to modulate the electrical field without causing thermal coagulation of tissue and risk of thermal tissue injury [29]. PRF generates two 20-millisecond pulses of RF waves every 0.5 seconds at a temperature carefully controlled below 42°C [24]. Pathophysiology of pain relief from PRF is debated; however, it is thought that electrical fields generated by PRF can affect neuronal membranes and alter c-Fos pain pathways decreasing the perception of pain [24].

Both CRF and PRF are used for the treatment of PMPS when conventional therapy fails, with current literature demonstrating efficacy for both. Only one study has compared CRF versus PRF for PMPS patients, which showed a greater decrease in VAS pain scores in patients not responding to oxycodone and pregabalin for at least four weeks in those treated with CRF compared to PRF of the stellate ganglion at all times points considered [27,30]. CRF thoracic sympathectomy was compared against sham in PMPS patients in 2020 showing significantly reduced VAS pain scores in the CRF group as well as decreased need for antineuropathic drugs and opioids up to six months post-procedure [27].

PRF has been demonstrated as an effective PMPS treatment as well. The first case report of PRF use in PMPS in 2010 showed successful pain relief in a patient with three years of chronic pain post-mastectomy [29]. A clinical trial analyzing PRF to the dorsal root ganglion (DRG) followed by steroid injection with dexamethasone and bupivacaine for four successive treatments, repeated weekly, decreased VAS pain scores and opioid consumption up to six months following the procedure [26]. Lastly, a RCT comparing PRF of the thoracic DRG versus PRF of the thoracic paravertebral nerve for PMPS pain showed success of both treatments in the reduction of pain; however, PRF at the DRG provided better long-term analgesia at six months [27,30].

Patient selection plays a major role in determining good candidates for radiofrequency ablation. Apart from usual considerations in patient selection, good candidates for RFA are also those for whom a diagnostic thoracic block has been successful in providing pain relief. These blocks are traditionally performed with lidocaine with or without steroids, and sustained pain relief is a key predictor in determining whether or not ablation of the nerve root would provide a long-term positive outcome [26-28,30].

Absolute contraindications to RF include increased intracranial pressure and local infection. Proximity to the spinal column is an important consideration as the risk of both infection and bleeding needs to be weighed in the decision-making process [24,26]. Among complications of RF treatment, periprocedural and postprocedural discomfort along with post-procedure neuropathic pain are most commonly reported. Although no specific studies have investigated the specific incidence of adverse effects in PMPS, literature estimates complications from RF therapy in patients undergoing breast-conserving surgery with RF for stage 1 breast cancer at less than 6% with skin burns being the most common (incidence of 0%-6%) with the procedure generally being well tolerated [24,28].

In summary, both CRF and PRF are valid options for the treatment of PMPS that fail first-line therapy. The only head-to-head trial comparing CRF and PRF techniques demonstrated superior chronic pain relief, decreased opioid consumption, and increased functional ability in the CRF group although a higher-powered study with more prolonged follow-up time is needed to corroborate these results. PRF generally has fewer adverse effects and rates of associated complications, justifying further exploration into its use for PMPS.

Neuromodulation

A more definitive and emerging approach over the last decade for the treatment of chronic pain syndromes is neuromodulation, a targeted electrical stimulation that alters peripheral and central nervous system neuronal firing patterns [31-34]. Traditional spinal cord stimulation (t-SCS) involves the development and modulation of electric fields between electrodes, which in turn leads to increased levels of pain-modulating neurotransmitters and suppression of hyper-excitable neurons that have been known to be drivers of neuropathic pain. Specifically, t-SCS targets large-diameter dorsal column neurons which in turn inhibit pain modulation and signaling within small-diameter neurons. There is also emerging evidence regarding the modulation of the descending pain pathway as well as the targeting of the emotional and affective pain components via the burst spinal cord stimulation technique [32]. T-SCS is often offered to patients whose pain syndromes are medically refractory or have shown some, temporary relief with nerve blocks or RFA. Especially in post-laminectomy syndrome and CRPS, t-SCS has shown significant promise and strong positive outcomes [33].

While the literature is limited in the success of traditional, dorsal column SCS in PMPS, there are case series available that highlight the significant promise in its utility [34,35]. A seven-patient case series done at a major US cancer center on PMPS showed 85% of patients achieving greater than 50% pain relief, with half of those getting greater than 75% relief as well [35]. Each of these patients had previously trialed multiple oral and regional anesthetic methods for pain relief, including intercostal nerve blocks and stellate ganglion blocks, with temporary, non-sustaining relief. Additionally, six out of the seven patients had significantly reduced pre- and post-oral morphine equivalent (OME) requirements, including two patients whose OME requirements went down to zero. Importantly, the patients in this study had a varying dermatomal distribution of their pain, going as high as C3 and as low as T8. The optimal lead placement was achieved through intraoperative patient feedback regarding the induction of paresthesia. Three of the patients had leads placed between C2-C5, three patients had leads placed between T1-T4, and one patient had a lead placed at C5 as well as another placed at T8. A promising aspect of the effectiveness of t-SCS in PMPS was the variation of symptoms that patients initially presented with, ranging from shooting arm and hand pain to spasms of the chest wall. As operative technique, degree of lymphedema, and burden of metastatic involvement can vary between patients, it is encouraging to see neuromodulation techniques impacting pain signals via a multitude of mechanisms.

Notably, one of the main driving factors for reduced levels of pain relief during the trial phase of SCS placement was the duration of PMPS symptoms. This is corroborated by multiple studies, which have highlighted a correlation between the duration of pain and the decreased reduction in pain relief. Further investigation with a larger patient base can help clarify this risk factor as an indicator of SCS success versus failure [33-35].

An alternative to t-SCS is DRG stimulation. Here, the electrical signals directly affect the primary cell bodies of the DRG [36,37]. The DRG is known to play a pivotal role in the development of neuropathic pain as it houses the somas of the primary sensory neurons and is a main link between the periphery and the central nervous system. In multiple studies, trials have shown promise for DRG stimulation as a more targeted and effective approach (increased root specificity) for pain relief as compared to t-SCS. The ACCURATE trial specifically showed greater pain relief, improved quality of life, decreased variation with postural changes, and less stimulation in nonpainful areas within the DRG cohort [36]. DRG stimulation at the thoracic level has a greater promise from accuracy due to difficulties in targeting tight dermatome levels for t-SCS as well as a thicker cerebrospinal fluid (CSF) layer (which can shunt some of the t-SCS electrical field energy.) While the literature on the utility of DRG in PMPS is relatively sparse, there are case reports and case series evaluating the utility of DRG stimulation in both PMPS as well as thoracic neuralgia (TN) [38,39].

An often-discussed case by Morgalla in Germany showed the utility of DRG stimulation in a patient with PMPS over four years. Pain scores dropped by four points on the NRS-11 scale, and the patient’s medication regimen decreased by 50% over the four years [39]. The main complication noted was local implantable pulse generator (IPG) site discomfort, but this was resolved with the relocation of the IPG. The patient was able to resume daily activities and return to work as well, with significant pain and functional improvement. An interesting six-patient case series on the use of DRG stimulation in chronic thoracic pain syndromes showed positive outcomes as well [34]. Patients in this case series had diagnoses ranging from unilateral and bilateral post-mastectomy pain to post-herpetic neuralgia to abdominoplasty. In this series, 33% converted from trial DRG stimulation to permanent implantation (all with postoperative pain - abdominoplasty and mastectomy). OME requirement in the successful cases was reduced to zero from 20-30 pre-implantation. VAS pain scores also went from as high as 8 down to as low as 0 in those two cases. This relief was sustained at the 18-month follow-up as well. The main reasons for which implantation was not pursued included pain caused by leads and lack of symptomatic relief. Of note, patients who did not successfully undergo permanent placement had higher baseline OMEs and longer duration of thoracic neuralgia before the DRG trial.

While the off-label use of DRG stimulation in thoracic pain syndromes in the United States is growing, DRG stimulation is currently only approved for pathology below the level of T-10 [36,38]. Due to the targeted nature of DRG stimulation, it may have increased utility in patients who have demonstrated response at specific levels of the thoracic spinal cord, e.g., via pulsed DRG radiofrequency ablation or peripheral nerve blocks. The most recent American Society of Pain and Neuroscience guidelines recommend DRG stimulation for breast and mastectomy-related pain between the levels of T3 and T7. Given that PMPS and other thoracic neuralgia syndromes have historically been difficult to control, DRG stimulation offers a promising, targeted, and safe option for refractory pain.

DRG stimulation has also shown promise as salvage therapy (i.e., when t-SCS, burst SCS, or other such modalities have failed) [40-42]. A retrospective review primarily focused on lower thoracic and lumbar cases was conducted on 60 patients who underwent salvage DRG stimulation and were followed for an average of three years. Overall, these patients showed greater than 55% improvement in pain scores and over 44% in disability index, suggesting that DRG stimulation should at least merit consideration in cases where t-SCS was not as effective [40]. While this study focused more on patients requiring lower thoracic and lumbar stimulation, further investigation is needed to evaluate the relative effectiveness of DRG stimulation as salvage therapy in upper-middle thoracic pain syndromes. We anticipate, over time, that the use of DRG stimulation will continue to rise, both as an alternative as well as a salvage therapy for thoracic pain pathology, including PMPS.

Peripheral nerve stimulation (PNS), is an alternative neuromodulation technique that has also been used in cases of cancer-related chest well pain. Here, leads are placed on spinal nerve roots (or other peripheral nerves) rather than on the dorsal column or DRG. Three case reports have been published regarding the use of PNS in PMPS, where the leads were placed on spinal nerve roots corresponding to the levels at which the pain was most predominant [43-45]. Two of the cases had the leads at the T2 and T4 roots, and the third had the leads at the C8 and T1 roots. In two cases, there was sustained improvement of pain scores from 10/10 down to 1/10, but in the third case, the device was ultimately not used by the patient due to rash at the sites of lead insertion.

Patient selection is key to the success of neuromodulation devices. For example, those who have well-localized pain syndromes, control of comorbidities such as diabetes and hypertension, good dietary habits, and the ability to avoid substances such as tobacco are predictors of neuromodulation success. Having good mental health support as well as the motivations and means for physical therapy and rehabilitation also lend to a higher likelihood of long-term success with neuromodulation. Adverse events commonly seen with SCS commonly include device malfunction, infection, pain at the implant site, and lead migration. Much less commonly seen are dural puncture/headache and neurological damage from SCS implantation. Overall, as technology and operative techniques continue to be refined, we anticipate that neuromodulation will become a more viable and sustainable option for a larger proportion of the PMPS population.

Intrathecal Drug Delivery System

Another effective pain management treatment option for those who have not responded well to more conservative treatments is intrathecal drug delivery systems. This approach allows for direct drug delivery to the dorsal horn of the spinal cord, enabling bypass of first-pass metabolism and lower overall effective doses. There is also less effect on systemic receptors, ensuring more targeted drug delivery and therapy. With the burden of opioid use in both cancer-related and non-cancer pain continuing to worsen, IDDS has shown significant promise in helping combat the epidemic. Guidelines regarding the safe and effective use of IDDS for chronic pain were established by the Polyanalgesic Consensus Conference (PACC) in 2016. Medications that were previously often in IDDS include bupivacaine, dilaudid, morphine, fentanyl, baclofen, and clonidine [46,47]. Specifically, for cancer-related pain, ziconotide has shown increasing promise as a potential intrathecal medication. Ziconotide, a non-opioid analgesic agent that acts as a reversible N-type calcium channel blocker, has been shown to prevent the presynaptic release of neurotransmitters, thereby preventing nociceptive signaling from going through. Randomized trials as well as retrospective reviews around the utility of Ziconotide have shown its efficacy in improving pain scores for both cancer and non-cancer-related pain by over 30% [46]. A retrospective review from the PRIZM study showed significantly improved pain relief when ziconotide was used as the first drug in pump (FIP) compared to when it was not used as the FIP [48]. This clinically significant improvement in pain scores and functionality was sustained at the 12-month follow-up period as well. Based on the PACC guidelines, IDDS with ziconotide was given a grade A recommendation for use in active cancer-related pain [46]. Specifically, when the sources of pain are well localized (e.g., T3-T7 for most cases of PMPS), IDDS is particularly effective. A study evaluating patient selection for IDDS with ziconotide echoed these sentiments. Their recommendation for the use of ziconotide hinged around two major characteristics: (1) predominantly neuropathic pain and (2) chronic pain refractory to systemic opioid use > 75 OME [46]. As many PMPS easily achieve this OME and neuropathic symptoms are an extremely common disease manifestation, the use of IDDS with ziconotide as FIP shows great promise in the PMPS population.

Discussion

**Figure 1:**
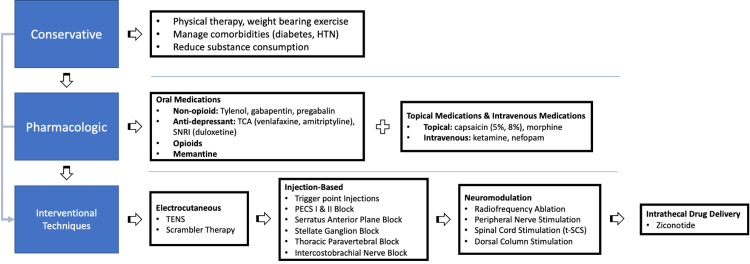
The suggested algorithm recommended for PMPS pain following the review of the current literature HTN: Hypertension; TCA: Tricyclic antidepressant; SNRI: Serotonin-norepinephrine reuptake inhibitors; TENS: Transcutaneous electrical nerve stimulation; PMPS: Post-mastectomy pain syndrome. Image credits: This image was created by the author, Jay D Shah.

Our review highlights a wide body of evidence to date on the management of chronic pain syndrome, which, while not as prevalent as some of the more commonly discussed conditions, has a significant disease burden and impact on the quality of life [49-52]. However, as with other common pain syndromes, treatment is best when individualized to the patient and their circumstance. Figure 1 highlights a reasonable treatment algorithm for managing PMPS as there have been many advances in recent years including the use of neuromodulation and intrathecal drug delivery systems. Historically, opioid analgesia has been a mainstay of both neuropathic and non-neuropathic pain. However, over the past 10 years, we have begun to establish more patient-centric treatment algorithms to limit the use of opioids while targeting the root cause of the pain pathway. The goal of such an algorithm is to help reduce patient opioid dependence and introduce interventional, opioid-sparing techniques of pain management early in the treatment experience of PMPS patients.

With any chronic pain syndrome due to surgery, the management is often thought to start during the perioperative period. The management of perioperative pain is beyond the scope of this review article, but several perioperative techniques are promising in reducing the effects of chronic pain. Most chronic pain physicians will often see these patients well beyond their perioperative period and require a deep understanding of the pathophysiology of the pain syndrome to better manage these patients aside from the use of only opioid medications. This article highlights several oral and topical analgesics that have promise in managing PMPS. If these medications fail to improve functional activity or quality of life, interventional techniques can be considered as an adjunct to pain management. The advent of more interventional and procedural techniques with supported data is especially important.

The type of pain is crucial in identifying the type of treatment best suited for the patient. Understanding the distinction between somatic, visceral, and neuropathic pain will help determine the effective treatment. As our article illustrates, the common theme in managing patients with PMPS is treating the underlying neuropathic pain. Antidepressants, gabapentinoids, and memantine have long been used for neuropathic pain with excellent relief. The primary use of interventional techniques such as trigger points, stellate ganglion, and thoracic blocks are diagnostic to help determine the type of pain. Patients responding to trigger point injections can help distinguish if the primary cause of pain is somatic or myofascial in nature. Patients responding to stellate ganglion blocks can consider neuropathic pain as the primary source. Patients responding to diagnostic thoracic blocks can consider the use of radiofrequency ablations as a treatment option. If these diagnostic and therapeutic treatment options fail to control the pain and it is determined the primary pain contributor is neuropathic pain, then the use of neuromodulation and intrathecal drug therapy has much promise.

Neuromodulation is very effective for neuropathic pain syndromes [33]. Patient selection is of utmost importance when considering to offer this treatment to patients. Patients should not have any severe underlying psychiatric condition and should be evaluated by a pain psychologist before proceeding with neuromodulation. In addition, we put great emphasis on the trial phase of the neuromodulation device. Not only should we see improvement in pain scores, but we should also see an improvement in sleep, function, mood, and activities of daily living. As neuromodulation has expanded with traditional dorsal column stimulation with various device companies and programs, DRG stimulation has had success with off-label use for PMPS and can be considered if dorsal column stimulation fails. Improved pain localization in PMPS is also a great sign for the future utility of PNS [32]. While discussed less in existing literature, it has a favorable outlook for PMPS as well due to its great safety profile, minimally invasive surgical approach, and ability to target specific nerve distributions. If these treatments fail, ziconotide for treating neuropathic pain can be considered with intrathecal drug delivery systems [47].

## Conclusions

Much of the current research dedicated to PMPS is limited to case reports and case series. Modalities discussed in this context lack blinding, leading to unavoidable bias. This review highlights the necessity of patient selection, pain-related factors, and the importance of a trial phase of neuromodulation when determining if a patient requires permanent implantation of an SCS. Limitations of this review include unavoidable heterogeneity in sample sizes and a lack of standardized outcome measures for comparison across studies. We believe that chronic pain after mastectomy has a much higher incidence than reported and recommend that further studies investigate the utility of interventional techniques using patient stratification to better understand the impact of interventional techniques in PMPS management.

## References

[1] Nardin S, Mora E, Varughese FM (2020). Breast cancer survivorship, quality of life, and late toxicities. Front Oncol.

[2] Waltho D, Rockwell G (2016). Post-breast surgery pain syndrome: establishing a consensus for the definition of post-mastectomy pain syndrome to provide a standardized clinical and research approach - a review of the literature and discussion. Can J Surg.

[3] Capuco A, Urits I, Orhurhu V (2020). A comprehensive review of the diagnosis, treatment, and management of postmastectomy pain syndrome. Curr Pain Headache Rep.

[4] Chappell AG, Yuksel S, Sasson DC, Wescott AB, Connor LM, Ellis MF (2021). Post-mastectomy pain syndrome: an up-to-date review of treatment outcomes. JPRAS Open.

[5] Gong Y, Tan Q, Qin Q, Wei C (2020). Prevalence of postmastectomy pain syndrome and associated risk factors: a large single-institution cohort study. Medicine (Baltimore).

[6] Yuksel SS, Chappell AG, Jackson BT, Wescott AB, Ellis MF (2022). "Post mastectomy pain syndrome: a systematic review of prevention modalities". JPRAS Open.

[7] Amr YM, Yousef AA (2010). Evaluation of efficacy of the perioperative administration of Venlafaxine or gabapentin on acute and chronic postmastectomy pain. Clin J Pain.

[8] Kalso E, Tasmuth T, Neuvonen PJ (1996). Amitriptyline effectively relieves neuropathic pain following treatment of breast cancer. Pain.

[9] Tasmuth T, Härtel B, Kalso E (2002). Venlafaxine in neuropathic pain following treatment of breast cancer. Eur J Pain.

[10] Belfer I, Pollock NI, Martin JL (2017). Effect of gastroretentive gabapentin (Gralise) on postmastectomy pain syndrome: a proof-of-principle open-label study. Pain Rep.

[11] Reyad RM, Omran AF, Abbas DN (2019). The possible preventive role of pregabalin in postmastectomy pain syndrome: a double-blinded randomized controlled trial. J Pain Symptom Manage.

[12] Vig S, Kumar V, Deo S (2019). Effect of perioperative pregabalin on incidence of chronic postmastectomy pain syndrome: a prospective randomized placebo-controlled pilot study. Indian J Palliat Care.

[13] Kreutzwiser D, Tawfic QA (2019). Expanding role of NMDA receptor antagonists in the management of pain. CNS Drugs.

[14] Morel V, Joly D, Villatte C (2016). Memantine before mastectomy prevents post-surgery pain: a randomized, blinded clinical trial in surgical patients. PLoS One.

[15] Watson CP, Evans RJ. (1992). The postmastectomy pain syndrome and topical capsaicin: a randomized trial. Pain.

[16] Marineo G, Iorno V, Gandini C, Moschini V, Smith TJ (2012). Scrambler therapy may relieve chronic neuropathic pain more effectively than guideline-based drug management: results of a pilot, randomized, controlled trial. J Pain Symptom Manage.

[17] Smith TJ, Wang EJ, Loprinzi CL (2023). Cutaneous electroanalgesia for relief of chronic and neuropathic pain. N Engl J Med.

[18] Khoury AL, Keane H, Varghese F (2021). Trigger point injection for post-mastectomy pain: a simple intervention with high rate of long-term relief. NPJ Breast Cancer.

[19] Abbas DN, Reyad RM (2018). Thermal versus super voltage pulsed radiofrequency of stellate ganglion in post-mastectomy neuropathic pain syndrome: a prospective randomized trial. Pain Physician.

[20] Elias M (2000). Cervical sympathetic and stellate ganglion blocks. Pain Physician.

[21] Dayem OTA, Saeid MM, Ismail OM (2014). Ultrasound guided stellate ganglion block in postmastectomy pain syndrome: a comparison of ketamine versus morphine as adjuvant to bupivacaine. J Anesth.

[22] Nabil Abbas D, Abd El Ghafar EM, Ibrahim WA, Omran AF (2011). Fluoroscopic stellate ganglion block for postmastectomy pain: a comparison of the classic anterior approach and the oblique approach. Clin J Pain.

[23] Kirkpatrick K, Khan MH, Deng Y, Shah KB (2023). A review of stellate ganglion block as an adjunctive treatment modality. Cureus.

[24] Wray JK, Dixon B, Przkora R (2024). Radiofrequency Ablation. https://www.ncbi.nlm.nih.gov/books/NBK482387/.

[25] Byrd D, Mackey S (2008). Pulsed radiofrequency for chronic pain. Curr Pain Headache Rep.

[26] Fam BN, El-Sayed G (2018). Efficacy and safety of pulsed radiofrequency and steroid injection for intercostobrachial neuralgia in postmastectomy pain syndrome—a clinical trial. Saudi J Anaesth.

[27] Hetta DF, Mohamed SAB, Mohamed KH (2020). Pulsed radiofrequency on thoracic dorsal root ganglion versus thoracic paravertebral nerve for chronic postmastectomy pain, a randomized trial: 6-month results. Pain Physician.

[28] Imoto S, Wada N, Sakemura N, Hasebe T, Murata Y (2009). Feasibility study on radiofrequency ablation followed by partial mastectomy for stage I breast cancer patients. Breast.

[29] Kim HT, Kim KY, Kim YD, Moon HS (2010). Pulsed radiofrequency lesioning for treatment of chronic breast neuropathic pain after breast reduction -a case report-. Korean J Anesthesiol.

[30] Van Zundert J, de Louw AJ, Joosten EA (2005). Pulsed and continuous radiofrequency current adjacent to the cervical dorsal root ganglion of the rat induces late cellular activity in the dorsal horn. Anesthesiology.

[31] Hagedorn JM, Pittelkow TP, Hunt CL, D'Souza RS, Lamer TJ (2020). Current perspectives on spinal cord stimulation for the treatment of cancer pain. J Pain Res.

[32] Abdullah NM, Jenkinson RH, Deer TR (2023). Treatment of chest wall pain syndrome from oncologic etiology with neuromodulation: a narrative review. Interv Pain Med.

[33] Dones I, Levi V (2018). Spinal cord stimulation for neuropathic pain: current trends and future applications. Brain Sci.

[34] Anthony CL, Tora MS, Bentley JN, Texakalidis P, Boulis NM (2019). Dorsal root ganglion stimulation for thoracic neuralgia: a report of six cases. Cureus.

[35] Matthew C, Ray H, Chen TH, Chen Y, Ansoanuur G, Le-Short C (2021). Treatment of postmastectomy pain syndrome with spinal cord stimulation: a case series. Pain Med Case Rep.

[36] Chapman KB, Sayed D, Lamer T (2023). Best practices for dorsal root ganglion stimulation for chronic pain: guidelines from the American society of pain and neuroscience. J Pain Res.

[37] Hong SW, Kim MJ, Park CH, Park S, Kim JH (2021). Dorsal root ganglion stimulation combined with spinal cord stimulation for effective treatment of postherpetic neuralgia - a case report. Anesth Pain Med (Seoul).

[38] Yang A, Nadav D, Legler A, Chen GH, Hingula L, Puttanniah V, Gulati A (2021). An interventional pain algorithm for the treatment of postmastectomy pain syndrome: a single-center retrospective review. Pain Med.

[39] Morgalla MH (2019). Dorsal root ganglion stimulation for the treatment of persistent post-mastectomy pain: case report. Neuromodulation.

[40] Reddy RD, Moheimani R, Yu GG, Chakravarthy KV (2020). A review of clinical data on salvage therapy in spinal cord stimulation. Neuromodulation.

[41] Chapman KB, Spiegel MA, van Helmond N (2022). Dorsal root ganglion stimulation as a salvage therapy following failed spinal cord stimulation. Neuromodulation.

[42] Yang A, Hunter CW (2017). Dorsal root ganglion stimulation as a salvage treatment for complex regional pain syndrome refractory to dorsal column spinal cord stimulation: a case series. Neuromodulation.

[43] Mainkar O, Solla CA, Chen G, Legler A, Gulati A (2020). Pilot study in temporary peripheral nerve stimulation in oncologic pain. Neuromodulation.

[44] Muenchrath MM, Gilani SO, Christiansen SL (2022). Peripheral nerve stimulator placement for neuropathic pain due to brachial plexus invasion by lung cancer: case report. Interventional Pain Medicine.

[45] Mainkar O, Singh H, Gargya A, Lee J, Valimahomed A, Gulati A (2021). Ultrasound-guided peripheral nerve stimulation of cervical, thoracic, and lumbar spinal nerves for dermatomal pain: a case series. Neuromodulation.

[46] Deer TR, Pope JE, Hanes MC, McDowell GC (2019). Intrathecal therapy for chronic pain: a review of morphine and ziconotide as firstline options. Pain Med.

[47] Pope JE, Deer TR, Bruel BM, Falowski S (2016). Clinical uses of intrathecal therapy and its placement in the pain care algorithm. Pain Pract.

[48] Deer T, Rauck RL, Kim P (2018). Effectiveness and safety of intrathecal ziconotide: interim analysis of the patient registry of intrathecal ziconotide management (PRIZM). Pain Pract.

[49] Poleshuck EL, Katz J, Andrus CH, Hogan LA, Jung BF, Kulick DI, Dworkin RH (2006). Risk factors for chronic pain following breast cancer surgery: a prospective study. J Pain.

[50] Rogowsky L, Illmann CF, Macadam SA (2022). Prevalence and severity of chronic pain in patients receiving mastectomy with alloplastic immediate breast reconstruction: a survey study [IN PRESS]. Plastic Surgery.

[51] Tan PY, Anand SP, Chan D (2022). Post-mastectomy pain syndrome: a timely review of its predisposing factors and current approaches to treatment. Proceedings of Singapore Healthcare.

[52] Tasmuth T, Kataja M, Blomqvist C, von Smitten K, Kalso E (1997). Treatment-related factors predisposing to chronic pain in patients with breast cancer--a multivariate approach. Acta Oncol.

